# Hepatitis B vaccination and post-exposure prophylaxis in healthcare workers – a comparative analysis of national and supranational recommendations

**DOI:** 10.3205/dgkh000647

**Published:** 2026-05-06

**Authors:** Daniela Ciochina, Johanna Stranzinger, Albert Nienhaus, Roland Diel

**Affiliations:** 1 Institute for Health Service Research in Dermatology and Nursing (IVDP), University Medical Center Hamburg-Eppendorf, Hamburg, Germany; 2 Institution for Statutory Accident Insurance and Prevention in the Health and Welfare Services (BGW), Hamburg, Germany; 3 Institute of Epidemiology, University Medical Hospital Schleswig-Holstein, Kiel, Germany

## Abstract

**Background:**

Needlestick injuries (NSIs) among healthcare workers (HCWs) remain common, with approximately 50,000 cases reported annually in Germany alone. Assessment of hepatitis B immune status is essential for post-exposure prophylaxis (PEP) following occupational exposure. However, hepatitis B vaccination coverage among HCWs in Europe is incompletely documented and varies substantially between countries.

**Objective:**

This review compares national and supranational recommendations regarding hepatitis B vaccination of HCWs, focusing on immune status monitoring and post-exposure prophylaxis after occupational exposure.

**Methods:**

A structured search of PubMed was conducted alongside a targeted review of official websites of national health authorities and professional societies in 16 European and Anglo-American countries, as well as recommendations issued by the World Health Organization (WHO) and the European Centre for Disease Prevention and Control (ECDC). National guidelines addressing occupational hepatitis B vaccination and PEP were identified and systematically compared.

**Results:**

Substantial differences exist between recommendations, particularly regarding the protective anti-HBs threshold, the definition and management of “non-responders”, and PEP strategies. Mandatory hepatitis B vaccination for exposed HCWs is in place in France and Belgium, as well as in selected states of the USA and Australia. While most countries closely follow the recommendations of the US Centers for Disease Control and Prevention (CDC), Germany, Austria, and Switzerland occupy a special position due to either a time-based criterion (10 years) or a tenfold higher protective threshold (anti-HBs ≥100 IU/L). Notable differences are also observed in PEP strategies, even among previously successfully vaccinated individuals.

**Conclusions:**

Although national recommendations share the common goal of preventing occupational HBV infection, considerable international variation persists. Given the favourable epidemiological trends and extensive experience with hepatitis B vaccination, harmonization of vaccination policies and post-exposure prophylaxis strategies would be desirable to improve clarity and strengthen occupational infection prevention across healthcare systems.

## Zusammenfassung

**Hintergrund:**

Nadelstichverletzungen (NSV) bei medizinischem Personal (MP) sind weiterhin häufig; allein in Deutschland werden jährlich etwa 50.000 Fälle gemeldet. Die Beurteilung des Hepatitis-B-Immunstatus ist für die Postexpositionsprophylaxe (PEP) nach beruflicher Exposition essenziell. Die Hepatitis-B-Impfquote unter MP in Europa ist jedoch nur unvollständig dokumentiert und variiert erheblich zwischen einzelnen Ländern.

**Zielsetzung:**

Dieser Review vergleicht nationale und supranationale Empfehlungen zur Hepatitis-B-Impfung von MP, mit Schwerpunkt auf der Überwachung des Immunstatus und der PEP nach beruflicher Exposition.

**Methoden:**

Es wurde eine strukturierte PubMed-Recherche durchgeführt, ergänzt durch eine gezielte Durchsicht offizieller Websites nationaler Gesundheitsbehörden und Fachgesellschaften aus 16 europäischen bzw. anglo-amerikanischen Ländern sowie der Empfehlungen der Weltgesundheitsorganisation (WHO) und des Europäischen Zentrums für die Prävention und Kontrolle von Krankheiten (ECDC). Nationale Leitlinien zur beruflich indizierten Hepatitis-B-Impfung und zur PEP wurden identifiziert und systematisch verglichen.

**Ergebnisse:**

Zwischen den Empfehlungen bestehen erhebliche Unterschiede, insbesondere hinsichtlich des protektiven anti-HBs-Grenzwerts, der Definition und des Managements von „Non-Respondern“ sowie der PEP-Strategien. Eine verpflichtende Hepatitis-B-Impfung für exponiertes MP besteht in Frankreich und Belgien sowie in einzelnen Bundesstaaten der USA und Australiens. Während die meisten Länder den Empfehlungen der US-amerikanischen Centers for Disease Control and Prevention (CDC) weitgehend folgen, nehmen Deutschland, Österreich und die Schweiz eine Sonderstellung ein – entweder aufgrund eines zeitbasierten Kriteriums (10 Jahre) oder eines zehnfach höheren protektiven Schwellenwerts (anti-HBs ≥100 IU/L). Bemerkenswerte Unterschiede zeigen sich auch bei den PEP-Strategien, selbst bei zuvor erfolgreich geimpften Personen.

**Schlussfolgerungen:**

Obwohl nationale Empfehlungen das gemeinsame Ziel verfolgen, berufsbedingte HBV-Infektionen zu verhindern, bestehen supranational erhebliche Unterschiede. Angesichts der günstigen epidemiologischen Entwicklungen und der langjährigen Erfahrung mit der Hepatitis-B-Impfung wäre eine Harmonisierung der Impfstrategien und der PEP wünschenswert, um die Nachvollziehbarkeit zu verbessern und die Prävention berufsbedingter Infektionen in Gesundheitssystemen zu stärken.

## Introduction

### Global and national epidemiology of hepatitis B

Hepatitis B virus (HBV) infection remains one of the most significant global public health challenges and is among the leading causes of infection-related mortality worldwide. In 2019, hepatitis B accounted for approximately 820,000 deaths, primarily due to liver cirrhosis and hepatocellular carcinoma. According to recent data from the World Health Organization (WHO) [1], an estimated 254 million people worldwide are currently living with chronic hepatitis B infection. The increase in hepatitis B-related deaths from 820,000 in 2019 to 1.1 million in 2022 has been attributed to several factors, including the ageing population of chronically infected individuals and disruptions to healthcare services during the COVID-19 pandemic, which reduced access to diagnosis and treatment in many low- and middle-income countries.

The European Centre for Disease Prevention and Control (ECDC) reported that in 2022 approximately 246,000 people in Germany were living with chronic hepatitis B infection, corresponding to a prevalence of around 0.3% of the total population [2]. Surveillance data submitted to the Robert Koch-Institute (RKI) show marked fluctuation in reported case numbers over recent years. Following a substantial decline during the first years of the COVID-19 pandemic (2020–2021), a sharp increase was observed in 2022 and 2023. In 2024, case numbers decreased again by approximately 5% compared with the previous year. These fluctuations are largely attributable to modified surveillance definitions, improved diagnostic practices, and migration-related factors rather than to a true increase in acute infections [3].

In younger birth cohorts vaccinated as part of routine immunization programs, the prevalence of hepatitis B is significantly lower than in older, unvaccinated or late-vaccinated cohorts, demonstrating the effectiveness of primary prevention through vaccination [3], [4].

### Occupational risk of HBV infection

Hepatitis B is a bloodborne viral infection. In Germany, the Standing Committee on Vaccination (STIKO) at the RKI defines occupational risk groups as individuals whose professional activities involve contact with human blood or other body fluids [5]. The most common transmission routes from infected patients to HCWs are needlestick injuries (NSIs) and other injuries caused by scalpels or sharp instruments, followed by mucocutaneous exposure. In Germany, approximately 50,000 NSIs are reported annually to the German Statutory Accident Insurance and Prevention in the Health and Welfare Services (BGW), and these numbers have remained consistently high across sectors [6].

Compared with other bloodborne pathogens such as hepatitis C virus and human immunodeficiency virus, HBV is considerably more transmissible in the healthcare setting. Individuals who are hepatitis B e antigen (HBeAg) positive exhibit high levels of infectivity [7], [8].

The introduction of hepatitis B vaccines in the 1980s represents the most important milestone in HBV prevention. Vaccination of HCWs and efforts to achieve high vaccination coverage constitute key components of strategies aimed at reducing nosocomial transmission. Post-exposure prophylaxis (PEP) following needlestick or other sharps injuries complement these preventive measures [9].

Under current European Union legislation, employers are required to conduct activity-based risk assessments to identify employees at risk of HBV exposure, inform them of the associated risks, and offer vaccination. Ideally, vaccination should be administered prior to or immediately after commencement of employment to prevent HBV infection and its long-term consequences, including chronic carrier status [10].

According to a WHO analysis, hepatitis B vaccination in countries with intermediate and high endemicity saves up to one hundred times the cost of vaccination per case of disease prevented [11]. Even in countries with low HBV prevalence, vaccination remains cost-effective [12].

In countries of the European Union and European Economic Area (EU/EEA), hepatitis B vaccination coverage among HCWs likely varies substantially. Comprehensive data are lacking and significant improvement is needed. In a 2024 ECDC survey, only eight countries were able to provide data on vaccination coverage among HCWs, with reported rates ranging from 15.1% to 100% [13].

The temporal trend of hepatitis B recognized as an occupational disease in Germany (occupational disease code BK-3101) shows a marked decline, particularly in recent years. Improved protective measures (e.g., safety-engineered devices) and increased vaccination coverage among HCWs have likely contributed to the reduction in occupationally acquired HBV infections [14]. In a 22-year time-trend analysis of hepatitis B among HCWs in Germany, Nienhaus et al. [15] demonstrated a decline in recognized occupational hepatitis B cases from 2.2 per 100,000 HCWs in 1996 to 0.2 per 100,000 in 2017.

The ECDC recommends hepatitis B vaccination for HCWs who may be exposed to blood or other potentially infectious materials as a priority measure to prevent occupational infection and to contribute to achieving the WHO global elimination targets. However, while the ECDC provides a strategic framework [16], detailed implementation remains the responsibility of national and international guidelines.

Existing literature on vaccination policies and PEP has so far been limited primarily to standardized surveys of vaccination experts from 21 [17] and 27 European Member States [18], focusing mainly on whether specific HCW groups are recommended for vaccination. This also applies to the ECDC report published in 2024 [12]. In these analyses, countries reported their policies, but the original texts of national recommendations were neither systematically retrieved nor cited. Consequently, a detailed content comparison has not previously been undertaken.

### Aim of the review

The aim is to identify and comparatively analyze national and supranational recommendations regarding pre-exposure hepatitis B vaccination and PEP for HCWs.

The following research questions were addressed:

- In which countries do written mandatory vaccination requirements for HCWs exist?
- Which countries recommend screening of HCWs for anti-HBs (with or without HBsAg and anti-HBc) prior to employment?
- In which countries is post-vaccination anti-HBs testing required, at what interval, and what threshold defines protective immunity?
- How are “low responders” and “non-responders” defined, and how are they managed?
- What PEP strategies are recommended for successfully vaccinated HCWs following needlestick injury, and how do these strategies differ?

## Methods

### Search strategy

National recommendations issued by health authorities or professional societies were identified using a combined search strategy consisting of a structured database search and a targeted web-based review. To minimize language barriers and ensure comparability, the following countries were included:

- Western European countries: Germany, France, Switzerland, Austria, the Netherlands, Belgium, Luxembourg. Denmark was included as a representative example of the Nordic countries within the Western European comparison group.
- Southern European countries: Spain, Portugal, Italy, Greece.
- Anglo-American countries: United Kingdom, Ireland, United States, Canada, Australia.

### Database search

A structured search was conducted in PubMed without language restrictions and without limitations regarding year of publication. The following search string was applied:

("hepatitis B vaccination"[tiab] OR ("hepatitis B"[tiab] AND "vaccination"[tiab]) 

OR "HBV vaccine"[tiab] OR "hepatitis B vaccine"[tiab])

AND

("healthcare workers"[tiab] OR "health personnel"[tiab] 

OR "occupational exposure"[tiab])

AND

("guideline"[tiab] OR "guidelines"[tiab] 

OR "recommendation"[tiab] OR "recommendations"[tiab])

### Targeted web-based search

Because national immunization and occupational health recommendations are frequently published outside indexed scientific journals, an additional structured manual search was performed on the official websites of national health authorities and governmental institutions in each country. Where necessary, structured search queries were used (e.g., “hepatitis B vaccination guideline site: gov.ch” or “site: gov.nl hepatitis B healthcare workers”) to locate relevant documents.

For each identified source, the following information was systematically documented:

- country,
- issuing institution,
- title of the document,
- year/version (if available),
- official web address.

Only original national recommendations with explicit reference to HCWs were included. Where required, non-German documents were translated into German using the professional translation software DeepL.com to allow structured comparison of content.

In addition to national guidelines, supranational recommendations from the ECDC, the WHO, and European consensus documents were included.

## Results

### Search

The PubMed search yielded 101 publications. However, only three documents were directly relevant to the study objective: two CDC recommendations from 2001 and 2002 [9], [19], and the European consensus recommendations by Puro et al. from 2005 [20].

In contrast, the manual web-based search identified official recommendations in all included countries except Luxembourg. In Luxembourg, reference is made to existing legislation, but no publicly available national guideline specifically addressing hepatitis B vaccination of HCWs or a structured PEP algorithm could be identified at the time of review.

In Belgium, although detailed vaccination mandates exist for HCWs, practical guidance on PEP refers to external sources, specifically the US CDC recommendations and the UK “Green Book” (see below).

At the supranational level, in addition to the European consensus recommendations [20], the WHO position paper on hepatitis B vaccination (2017) was identified as a relevant document [21].

### Mandatory vaccination versus recommendations for HCWs

In most of the countries included in this review, hepatitis B vaccination for HCWs is recommended but not mandatory (Table 1 (Tab. 1)). Only a small number of countries have introduced legally binding vaccination requirements.

France represents one of the most prominent examples of mandatory hepatitis B vaccination for healthcare personnel. This has been in place since 31 January 1992 under Article L3111-4 of the French Public Health Code. This regulation applies to occupational groups at risk of infection working in prevention, care, or institutions providing services to older people. The requirement was reaffirmed in an updated legal version published on 23 May 2017 [22].

Belgium has adopted a similar legal approach. Under the Royal Decree of 29 April 1999 (Arrêté Royal), vaccination is mandatory for workers with occupational exposure risk, whereas employees without direct contact with infectious material (e.g., administrative staff) are exempt. HCWs must either demonstrate immunity to hepatitis B or undergo vaccination. Within two months after completion of the primary vaccination series, the immune response must be verified. Occupational physicians are responsible for booster vaccinations and for managing incidents involving exposure to blood [23].

Outside continental Europe, mandatory or quasi-mandatory vaccination requirements are implemented through employment regulations rather than national legislation. In Australia, for example, vaccination policies are issued at the level of individual states. Health authorities in Queensland and New South Wales require documented vaccination or proof of immunity before employment in healthcare settings [24], [25]. Comparable regulations exist in several US states, including Arkansas, the District of Columbia, Illinois, Kansas, Michigan, Rhode Island, South Carolina, and Washington [26].

In Italy, according to a publication by Squeri et al. [27], hepatitis B vaccination is mandatory for HCWs in certain regions (Apulia, Emilia-Romagna, and Marche) for staff working in operational clinical areas. However, these claims could not be confirmed on official Italian governmental websites.

Overall, mandatory hepatitis B vaccination for HCWs remains the exception rather than the rule, with most countries relying on strong recommendations rather than legally binding obligations.

### Screening of HCWs for anti-HBs (and, where applicable, HBsAg and anti-HBc) prior to employment

Verification of hepatitis B vaccination status before the commencement of occupational activities associated with potential exposure is included in all reviewed recommendations. The following section therefore focuses on notable differences between countries.

The CDC recommend screening for HBsAg, anti-HBs, and anti-HBc prior to vaccination in certain settings. This approach aims to avoid unnecessary vaccination of individuals who are already immune as a result of previous infection or prior immunization [28].

In contrast, the European consensus recommendations consider routine pre-vaccination serological screening to be generally not indicated, primarily for reasons of cost-effectiveness and practicality [20]. Similarly, the WHO recommends screening only for HCWs who have not previously been vaccinated [21].

In the German S3 guideline on hepatitis B infection [29], it is recommended that the occupational health examination at the beginning of employment should exclude chronic or occult HBV infection by testing for HBsAg and anti-HBc, while immunity should be documented through anti-HBs testing. If immunity is absent, hepatitis B vaccination should be initiated and the immune response subsequently verified (target anti-HBs titre ≥100 IU/L).

In the United Kingdom, hepatitis B screening prior to employment is recommended primarily for HCWs performing exposure-prone procedures (EPPs). If HBsAg testing is negative, vaccination should be offered if not previously completed, and vaccine response should be confirmed by measurement of anti-HBs antibodies [30].

A distinctive approach exists in Switzerland, which applies a specific algorithm when HCWs start an occupation involving potential exposure. If a complete hepatitis B vaccination series was administered within the previous five years, but no anti-HBs titre was measured 4–8 weeks after the last dose, anti-HBs testing should be performed. If the most recent vaccine dose was administered five years or more previously, an additional booster dose should first be given, followed by anti-HBs testing four to eight weeks later [31].

### Post-vaccination Anti-HBs testing

For clarity, all anti-HBs titres reported in this review are expressed uniformly in international units per litre (IU/L), regardless of the notation used in the original recommendations (e.g. mIU/mL, IU/L or mIE/mL).

In all analysed countries, measurement of the anti-HBs antibody titre after hepatitis B vaccination is recommended for HCWs with potential exposure to blood or body fluids.

The CDC/ACIP recommendations regarding post-vaccination serological testing have evolved over time. Earlier guidance, such as the ACIP recommendations for immunization of health-care personnel published in 2011 [32], suggested that post-vaccination anti-HBs testing should primarily be performed for HCWs at increased occupational risk of exposure to blood or body fluids. For personnel with lower exposure risk, routine testing was considered unlikely to be cost-effective and could therefore depend on institutional policy. More recent CDC guidance has provided a more detailed and standardised framework for evaluating hepatitis B immunity among healthcare personnel and for post-exposure management [33]. Post-vaccination anti-HBs testing is also recommended in Australia, primarily for HCWs who have frequent occupational contact with human tissue, blood or body fluids [34].

In most countries, anti-HBs titres are measured four to eight weeks after completion of the vaccination series (Table 2 (Tab. 2)). Against this general pattern, several national guidelines specify different testing intervals. Italy recommends testing exactly one month after the final vaccine dose according to a ministerial decree [35], whereas Ireland recommends testing two months after completion of the vaccination series [36]. Austria recommends measuring the antibody response six months after vaccination, based on the observation that 5–10% of vaccinated individuals may initially fail to develop a sufficient immune response, although testing as early as four weeks after vaccination is also considered acceptable [37]. Canada allows a broader interval, recommending anti-HBs testing between one and six months after completion of the primary series [38].

### Periodic antibody testing, booster vaccination and protective thresholds

Routine periodic anti-HBs testing or booster vaccination after successful immunisation is recommended in only a few countries, and even there only under specific circumstances.

In Germany, the S3 guideline recommends that HCWs with particularly high occupational exposure risk (i.e. continuous or repeatedly occurring contact with potentially infectious blood) undergo anti-HBs testing every 10 years [29]. If the anti-HBs titre falls below 100 IU/L, a booster vaccination is recommended. Austria follows a broadly similar approach. For HCWs with documented anti-HBs titres ≥100 IU/L, booster vaccination is recommended every 10 years as long as occupational exposure risk persists, while routine interim antibody testing is not required [37].

The Swiss recommendations issued by the Federal Office of Public Health (FOPH) and the Federal Commission for Vaccination (EKIF) apply a more differentiated strategy for HCWs who were fully vaccinated but lack documentation of post-vaccination antibody testing [31]. If the last vaccine dose was administered less than five years earlier, anti-HBs should be measured directly and a booster vaccination is recommended if the antibody level is <100 IU/L. If the last vaccination occurred five years or more previously, a booster dose should first be administered, followed by anti-HBs testing four to eight weeks later. These procedures primarily apply when HCWs enter or change an occupation involving exposure risk.

In contrast, the United Kingdom recommends against booster vaccination when an anti-HBs titre ≥100 IU/L has been documented after primary immunisation. Earlier guidance suggesting booster doses every five years (e.g. in earlier editions of the Green Book) has been withdrawn in the most recent version [39]. However, if an anti-HBs level between 10 IU/L and 100 IU/L is identified at any time after primary vaccination, a single booster dose is recommended (“whenever identified, even if years later”).

Taken together, these recommendations illustrate two different approaches to long-term protection after successful hepatitis B vaccination among HCWs: some countries maintain periodic monitoring or booster strategies in individuals with ongoing occupational exposure risk, whereas others consider primary immunisation with documented antibody response to provide sufficiently durable protection without routine booster vaccination.

### Strategies for low- and non-responders after hepatitis b vaccination

The terms “low responder” and “non-responder” are not defined uniformly across international recommendations (Table 3 (Tab. 3)).

The definition of a non-responder as an individual with an anti-HBs titre <10 IU/L after hepatitis B vaccination originates from CDC recommendations published in 1997 [40]. According to this concept, a lack of vaccine response is confirmed only after failure to achieve protective antibody levels following a second complete vaccination series.

According to current CDC recommendations [41], as well as the national guidelines of Portugal [42], Spain [43], Greece [44], Canada [38], the United Kingdom [39], and Ireland [36], an individual is classified as a non-responder only if the anti-HBs titre remains <10 IU/L after two complete vaccination series (six doses in total).

The Australian Immunisation Handbook defines a non-responder as a person with anti-HBs <10 IU/L measured 4–8 weeks after the third vaccine dose, but still recommends completion of a second vaccination series, resulting in a total of six doses [34].

In the Danish recommendations, the term “non-responder” is not explicitly used, but the concept can be inferred. Individuals who do not develop a detectable antibody response after the standard vaccination schedule receive an additional dose. If the anti-HBs titre remains <10 IU/L four weeks later, two further doses may be administered at one month and six months, resulting in a 3+1+2 schedule. Serological testing is recommended after completion of the series, although explicit threshold values are not always specified [45], [46].

The Italian recommendations differ slightly in that additional vaccine doses are administered sequentially with serological testing after each dose. If anti-HBs remains <10 IU/L, up to seven vaccine doses in total may be administered [47].

The WHO position paper does not provide detailed guidance for the management of individuals with anti-HBs <10 IU/L after vaccination [21].

German recommendations diverge from the CDC concept. In an earlier STIKO recommendation from 2013, individuals with anti-HBs levels between 10 and 99 IU/L are classified as low responders [48]. In such cases, an additional vaccine dose is recommended immediately, followed by repeat antibody testing after 4–8 weeks. If the titre remains <100 IU/L, two further vaccine doses are recommended. Individuals with anti-HBs levels <10 IU/L are classified as non-responders, but the management strategy is essentially identical to that used for low responders, provided that active HBV infection has been excluded.

A similar approach is used in Switzerland, where recommendations distinguish between “hypo-responders” (anti-HBs <100 IU/L) and “non-responders” (anti-HBs <10 IU/L) [48]. According to the Swiss vaccination schedule [49], if anti-HBs remains <100 IU/L after three vaccine doses, an additional dose should be administered. If the titre remains <100 IU/L, serological testing for HBsAg and anti-HBc should be performed to exclude infection. If infection is ruled out, additional doses may be administered at intervals of 2–6 months, up to a total of six doses, until an antibody level ≥100 IU/L is achieved. If this threshold is still not reached after six doses, further vaccination decisions are made on an individual basis, potentially including the use of combined vaccines or higher antigen doses.

The Austrian recommendations distinguish between low responders (anti-HBs <20 IU/L) and non-responders (no measurable antibody response, below the limit of detection). Individuals with titres between 20 and 99 IU/L receive an additional vaccine dose followed by repeat testing. If the titre remains below the protective threshold, further vaccination is recommended. For titres <20 IU/L or absent antibody response, vaccination with higher antigen content (e.g. double dose or an alternative vaccine preparation) may be considered [37].

The Dutch recommendations distinguish between “zero responders” (no detectable anti-HBs antibodies, below the limit of detection) and “poor responders” (detectable but <10 IU/L). Poor responders receive a booster vaccination followed by repeat antibody testing; if antibody levels remain insufficient, two additional doses are administered. Zero responders are first retested after the third dose. If the anti-HBs titre remains <10 IU/L after six doses, further vaccination is not recommended [50].

In France, recommendations largely follow the European consensus guidelines of Puro et al. [20]. These recommendations are notable for suggesting serological testing even before vaccination in unvaccinated individuals. According to these guidelines, individuals with anti-HBs <10 IU/L after the first vaccination series are considered non-responders. If this persists after six vaccine doses, additional strategies may be considered. These include the use of combined vaccines, double-dose formulations, or vaccines containing additional viral antigens (S, Pre-S1, and Pre-S2) such as Sci-B-Vac^®^ or GeneVac-B^®^. Such vaccines are used in some regions (e.g. Asia) but are not licensed in most European countries, including Germany [51].

### Post-Exposure Prophylaxis (PEP)

In the WHO position paper on hepatitis B vaccination (2017), PEP is discussed primarily in the context of newborns of HBsAg-positive mothers, recommend-ding rapid administration of hepatitis B immunoglobulin (HBIG), ideally within 24 hours after birth [21]. Occupational exposures such as needlestick injuries among HCWs are not addressed in detail.

In contrast, national recommendations provide more detailed guidance for post-exposure management following NSIs. As with recommendations on antibody monitoring, substantial differences exist between countries. The following comparison focuses specifically on PEP strategies for HCWs who were previously successfully vaccinated.

**Figure 1:**
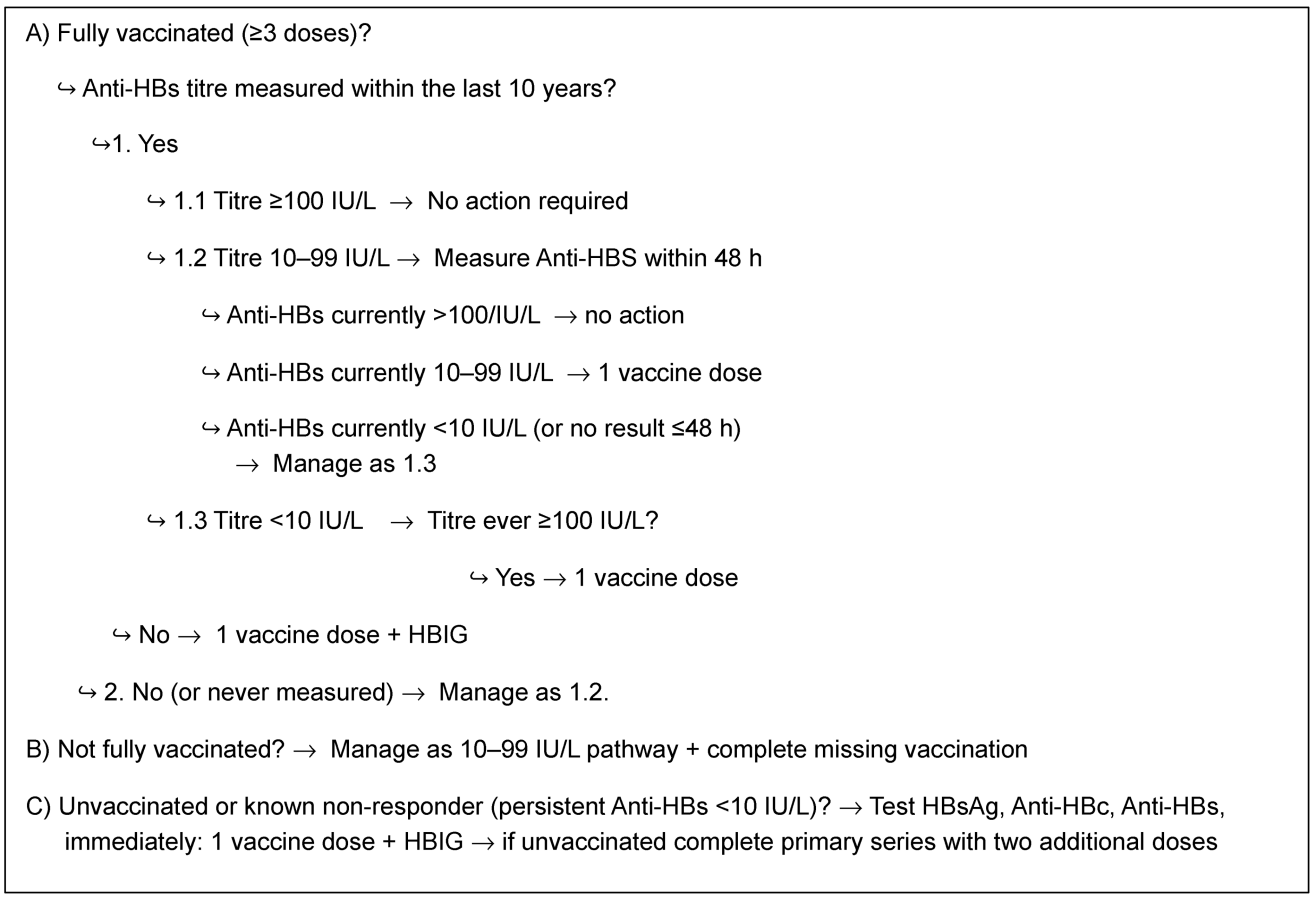
Decision tree of the German STIKO 2024 recommendations for hepatitis B post-exposure prophylaxis (modified from [5])

According to the German STIKO recommendations [5], management following exposure to an HBsAg-positive or unknown source patient is determined by three factors: the time elapsed since vaccination (≤10 years vs >10 years), the documented post-vaccination antibody response (anti-HBs ≥100 IU/L), and the availability of a current anti-HBs test result within 48 hours. The decision algorithm is illustrated in Figure 1 (Fig. 1). If an anti-HBs titre ≥100 IU/L has been documented within the previous 10 years, no further measures are required. If the last vaccination or antibody measurement occurred more than 10 years previously, anti-HBs testing should be performed immediately; if the current titre is ≥100 IU/L, no further action is necessary, whereas a titre of 10–99 IU/L indicates the need for a single booster dose and a titre <10 IU/L requires both a booster vaccination and administration of HBIG. 

The Austrian recommendations [37], similar to the German guidelines, place strong emphasis on the 10-year interval since vaccination. If a previously documented protective antibody level (anti-HBs ≥100 IU/L) is available but vaccination occurred more than 10 years prior, the current anti-HBs level should be measured. If the titre remains ≥100 IU/L, no intervention is required. However, differences arise versus the German recommendations in cases in which the antibody level is <100 IU/L: titres of 20–99 IU/L are managed with a single booster vaccination, whereas titres <20 IU/L (in Germany: <10 IU/L) lead to the recommendation of additional vaccine doses rather than administration of HBIG.

Unlike the German and Austrian recommendations, the Swiss guidelines do not include a time-based criterion. Time elapsed since a fully vaccinated individual’s anti-HBs titre ≥100 IU/L was last documented is irrelevant. No action is required after exposure [49]. Despite this, the same protective anti-HBs cut-off value (≥100 IU/L) is recommended as in the German and Austrian guidelines.

The Dutch national guideline on needlestick injuries (Landelijke Richtlijn Prikaccidenten) defines a protective anti-HBs threshold of ≥10 IU/L, which is considerably lower than the threshold used in German-speaking countries [52]. Like Switzerland, the Netherlands apply no time criterion. Of note, these recommendations differ from most other national guidelines in that post-exposure management depends not only on the HBV status of the source patient, but also on the type and severity of the exposure (e.g. low-risk vs high-risk injury). However, these considerations mainly affect individuals who are incompletely vaccinated or vaccinated without documented antibody response.

In the Irish guidelines [36], a similar consideration of the severity of exposure is applied. Even in fully vaccinated HCWs with documented protective antibody levels (anti-HBs ≥10 IU/L), administration of an additional vaccine dose may be considered in cases of severe exposure (e.g. deep needlestick injury or human bite) if the source patient is HBsAg positive.

According to the CDC recommendations [32], [33], which form the basis for many national guidelines, no post-exposure prophylaxis is required for HCWs with documented anti-HBs levels ≥10 IU/L, regardless of the time elapsed since vaccination, or the type of occupational exposure. This approach has been adopted by several countries, including Australia [34], Canada [38], [53], the United Kingdom [39], Portugal [42], Spain [43], and Greece [44].

In Belgium, official public health websites do not provide a dedicated guideline for hepatitis B vaccination of HCWs. However, the central information portal of the Belgian public health authorities refers explicitly to both the CDC recommendations and the UK “Green Book” as sources of guidance [54].

At the level of previously successful vaccination with a documented antibody response, the European consensus recommendations proposed by Puro et al. [20] are essentially identical to the CDC recommendations and are reflected, either explicitly or implicitly, in several national guidelines. The website of the French Ministry of Health explicitly refers to these European consensus recommendations for the management of occupational hepatitis B exposure among HCWs [55]. The Italian recommendations by Puro et al. (2003) [47] are largely identical to the later European consensus recommendations published in 2005 [20] and can be considered their conceptual basis. Similarly, the Danish recommendations state that HCWs who were vaccinated against hepatitis B and demonstrated an antibody response ≥10 IU/L after vaccination require neither testing nor additional vaccination following exposure [45], [46].

## Discussion

The present study provides, to our knowledge, the first systematic and detailed comparative overview of hepatitis B vaccination policies and PEP strategies for HCWs across 16 European and Anglo-American countries. A clear influence of the CDC recommendations is evident, as many national guidelines are closely aligned with the CDC framework. However, recent structural changes affecting US federal public health institutions may potentially alter the future role of the CDC as a global reference authority.

Where national policies diverge from the CDC framework, considerable heterogeneity becomes apparent, particularly within Western Europe. Only France [22] and Belgium [23] have established mandatory hepatitis B vaccination for HCWs. In most other Western European countries, vaccination is strongly recommended but remains voluntary. The lack of standardisation within Europe complicates cross-country comparisons and may contribute to regional gaps in vaccination coverage. Notably, even countries with relatively high hepatitis B prevalence, such as Greece or Italy, do not impose nationwide vaccination mandates for HCWs.

Regarding serological screening prior to employment, the comprehensive strategy recommended in Germany [29] and Switzerland [31]—including HBsAg, anti-HBc, and anti-HBs—is exceptional. By enabling the early identification of both non-responders and chronically infected individuals, this approach reduces the risk of nosocomial transmission, yet it is seldom adopted as a standard elsewhere.

One of the most striking differences between national recommendations concerns the protective antibody threshold. While Germany [29], Switzerland [31], and Austria [36] require an anti-HBs titre of ≥100 IU/L, most other countries consider ≥10 IU/L sufficient, in accordance with CDC recommendations [33]. The higher threshold used in German-speaking countries is justified by the assumption of improved long-term protection and reduced risk in the context of post-exposure management. However, robust evidence demonstrating superior long-term protection associated with this higher threshold remains limited.

Routine periodic antibody testing or booster vaccination after successful immunisation is recommended in only a few countries. In Germany and Austria, repeat testing or booster vaccination may be recommended after 10 years, particularly for individuals with ongoing occupational exposure risk. In contrast, most other countries rely on the durability of the primary vaccination series without routine follow-up testing. Considering the substantial inter-individual variability in immune response and antibody persistence, periodic monitoring in high-risk occupational settings may be a reasonable approach, although it inevitably increases logistical complexity and costs.

The definition and management of low and non-responders also vary considerably between countries. Most countries follow the CDC concept defining non-responders as individuals with anti-HBs levels <10 IU/L after two complete vaccination series. In contrast, Germany, Austria, and Switzerland apply alternative definitions or introduce additional categories such as hypo-responders or zero responders, reflecting a more differentiated immunological interpretation of vaccination response.

The largest international differences were observed in recommendations for post-exposure prophylaxis (PEP). In most countries, no intervention is recommended for HCWs with documented protective immunity after vaccination. However, variability exists even within this group, particularly regarding the threshold considered protective (10 IU/L vs 100 IU/L), the management of lower antibody levels detected years after vaccination, and the indications for HBIG administration.

In contrast to the CDC-based recommendations and most European guidelines, Germany and Austria employ more complex decision algorithms incorporating factors such as historical antibody titres, vaccination history, and time elapsed since immunisation. While such differentiated algorithms may theoretically improve risk stratification, they also increase complexity in clinical decision-making.

## Conclusion

Despite a broadly shared objective—namely the prevention of hepatitis B virus infection among HCWs—national recommendations regarding hepatitis B vaccination and PEP remain strongly country-specific. Differences exist regarding vaccination mandates, pre-employment screening strategies, protective antibody thresholds, definitions and management of vaccine non-response, and particularly in the algorithms applied for post-exposure prophylaxis.

While many national guidelines follow the general framework established by the CDC, notable deviations are observed in several European countries, especially in Germany, Austria, and Switzerland, where higher protective antibody thresholds and more differentiated post-exposure algorithms are applied.

Given the favourable epidemiological development of hepatitis B in many countries and the extensive global experience with hepatitis B vaccination, a discussion on the harmonisation of recommendations appears warranted. Greater alignment of vaccination policies and PEP strategies could improve clarity and facilitate occupational health decision-making for healthcare institutions and occupational health services. For the European region, the development of a comprehensive ECDC guideline addressing hepatitis B vaccination and post-exposure management in HCWs could therefore represent an important step, not only from a public health perspective but also in terms of healthcare system efficiency and occupational safety.

## Notes

### Author’s ORCIDs

- Diel R: https://orcid.org/0000-0001-8304-7709
- Nienhaus A: https://orcid.org/0000-0003-1881-7302

### Funding

No funding was received for this study.

### Competing interests

The authors declare that they have no competing interests.

## Figures and Tables

**Table 1:**
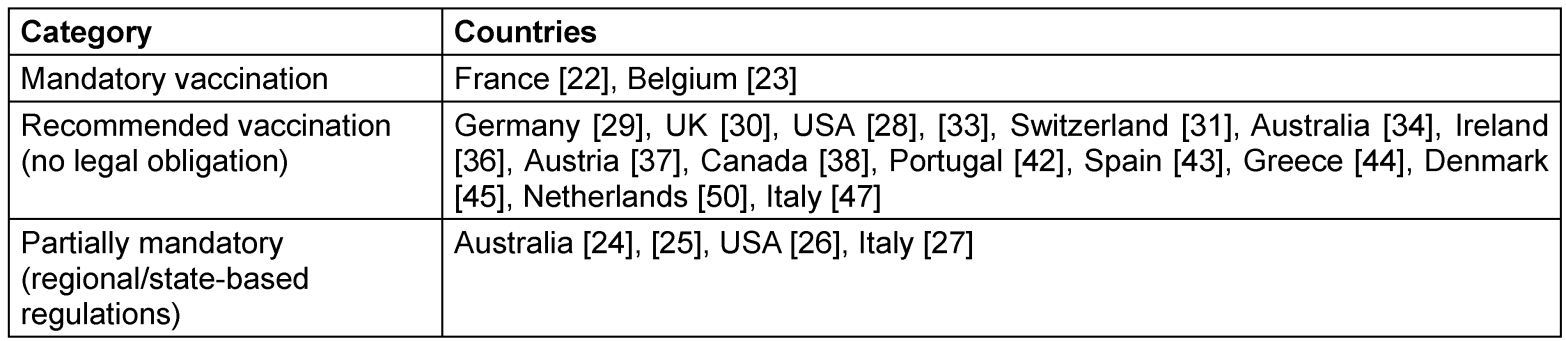
Countries by vaccination policy for HCWs

**Table 2:**
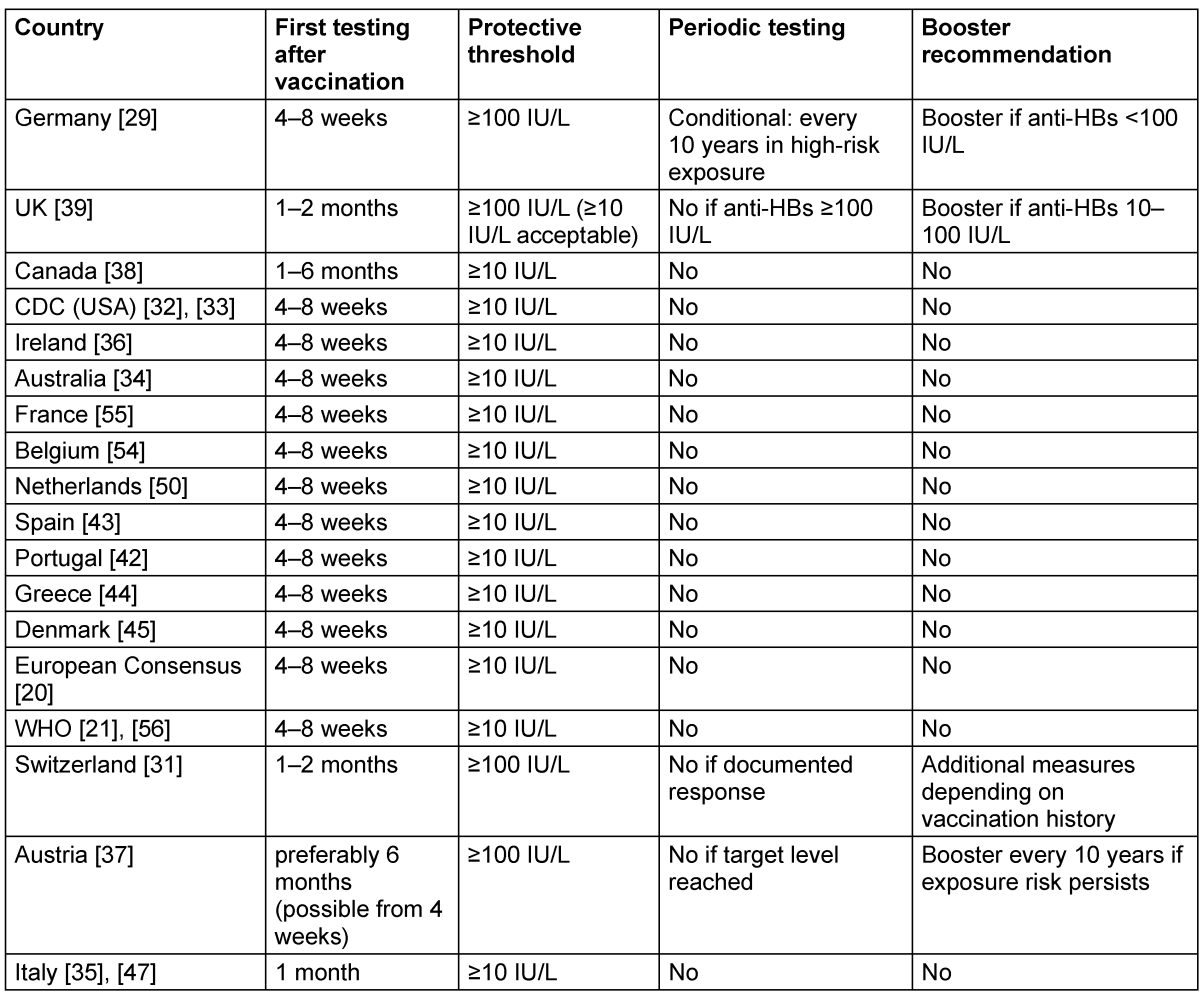
Post-vaccination antibody testing, protective thresholds and booster recommendations

**Table 3:**
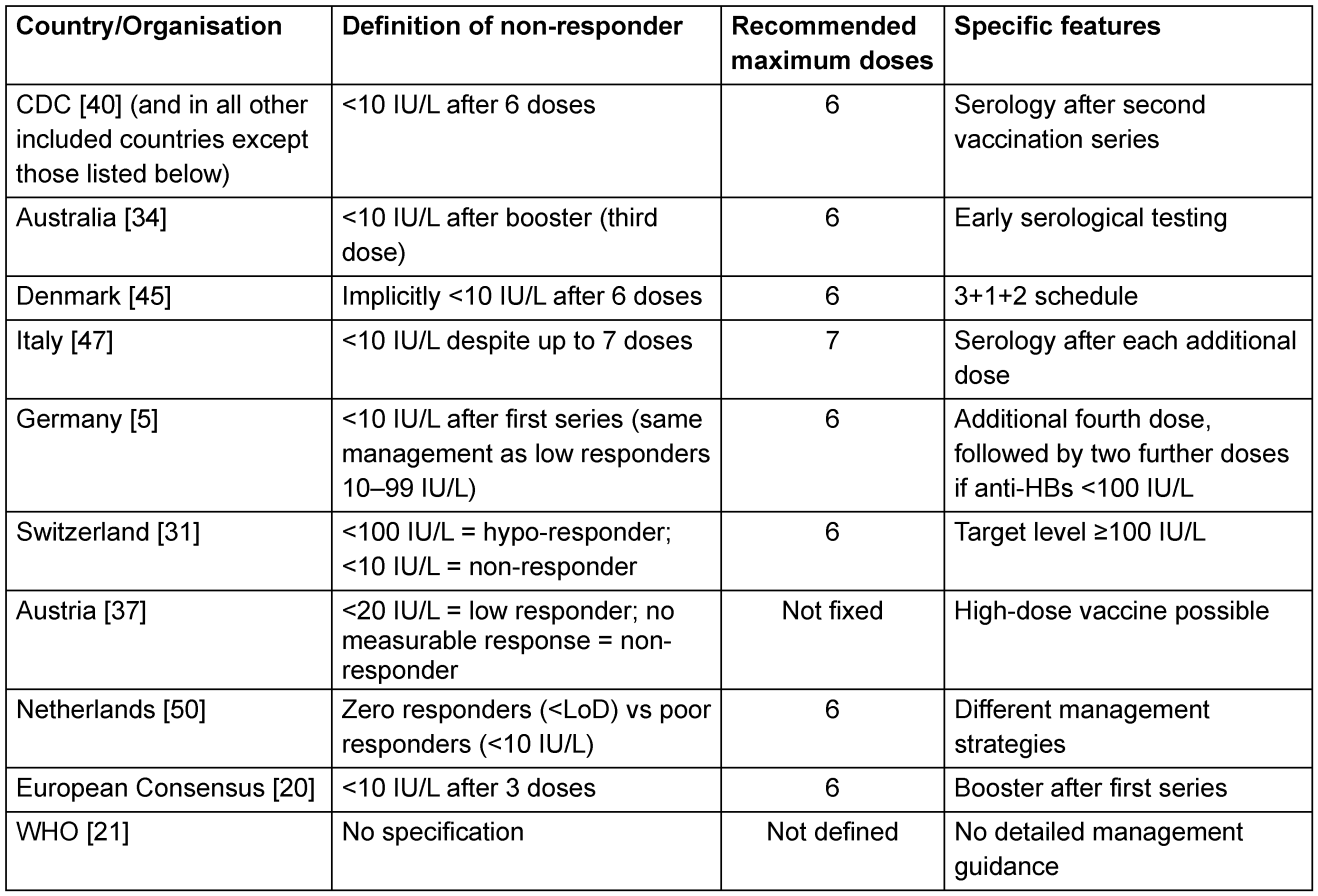
Comparison of definitions and management of non-responders
